# Correction: Kim et al. Neuroprotective Effect of Statins in a Rat Model of Chronic Ocular Hypertension. *Int. J. Mol. Sci.* 2021, *22*, 12500

**DOI:** 10.3390/ijms251910457

**Published:** 2024-09-27

**Authors:** Mi-Lyang Kim, Kyung Rim Sung, Junki Kwon, Go Woon Choi, Jin-A Shin

**Affiliations:** 1 Biomedical Research Center, College of Medicine, University of Ulsan, Asan Medical Center, Seoul 05505, Republic of Korea; iota76@gmail.com (M.-L.K.); goni8586@gmail.com (G.W.C.); jina7316@naver.com (J.-A.S.); 2 Department of Ophthalmology, College of Medicine, University of Ulsan, Asan Medical Center, Seoul 05505, Republic of Korea; ssazzang1982@hanmail.net

In the original publication [1], there was a mistake in Figure 3. Simvastatin protects RGCs from chronic OHT-induced cell death as published. The incorrect H&E image was placed mistakenly. The corrected Figure 3 appears below. The author Jin A Shin’s name has been changed to “Jin-A Shin”. The authors apologize for any inconvenience caused and state that the scientific conclusions are unaffected. The original publication has also been updated.




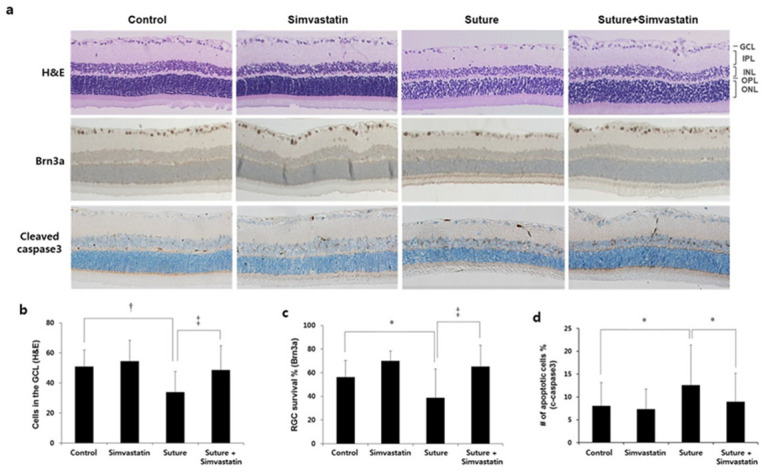




## References

[1] Kim M.-L., Sung K.R., Kwon J., Choi G.W., Shin J.-A. (2021). Neuroprotective Effect of Statins in a Rat Model of Chronic Ocular Hypertension. Int. J. Mol. Sci..

